# Characterizing non-communicable disease trends in undocumented migrants over a period of 10 years in Italy

**DOI:** 10.1038/s41598-023-34572-3

**Published:** 2023-05-08

**Authors:** Gianfrancesco Fiorini, Matteo Franchi, Giacomo Pellegrini, Antonello Emilio Rigamonti, Alessandro Sartorio, Nicoletta Marazzi, Giovanni Corrao, Silvano Gabriele Cella

**Affiliations:** 1 Istituti Clinici Zucchi, GSD, Monza, Italy; 2 National Centre for Healthcare Research and Pharmacoepidemiology, Milan, Italy; 3 Laboratory of Healthcare Research and Pharmacoepidemiology, Department of Statistics and Quantitative Methods, University of Milano Bicocca, Milan, Italy; 4 Laboratory of Clinical Pharmacology and Pharmacoepidemiology, Department of Clinical Sciences and Community Health, University of Milano, Milan, Italy; 5 Experimental Laboratory for Auxo-Endocrinological Research, Istituto Auxologico Italiano, IRCCS, Milan, Italy

**Keywords:** Health care, Medical research

## Abstract

Undocumented migrants represent a large part of the population in Countries of the European Union (EU) such as Italy. Their health burden is not fully understood and likely to be related mainly to chronic conditions. Information on their health needs and conditions may help to target public health interventions but is not found in national public health databases. We conducted a retrospective observational study of non-communicable disease (NCD) burden and management in undocumented migrants receiving medical care from Opera San Francesco, a non-governmental organization (NGO) in Milan, Italy. We analyzed the health records of 53,683 clients over a period of 10 years and collected data on demographics, diagnosis and pharmacological treatments prescribed. 17,292 (32.2%) of clients had one or more NCD diagnosis. The proportion of clients suffering from at least one NCD increased from 2011 to 2020. The risk of having an NCD was lower in men than women (RR = 0.88, 95% CI 0.86–0.89), increased with age (*p* for trend < 0.001) and changed with ethnicity. African and Asian migrants had a lower risk than Europeans of cardiovascular diseases (RR 0.62 CI 0.58–0.67, RR 0.85 CI 0.78–0.92 respectively) and mental health disorders (RR 0.66 CI 0.61–0.71, RR 0.60 CI 0.54–0.67 respectively), while the risk was higher in Latin American people (RR 1.07 CI 1.01–1.13, RR 1.18 CI 1.11–1.25). There was a higher risk of diabetes in those from Asia and Latin America (RR 1.68 CI 1.44–1.97, RR 1.39 CI 1.21–1.60). Overall, migrants from Latin America had the greatest risk of chronic disease and this was true for diabetes, cardiovascular diseases and mental health disorders. Undocumented migrants demonstrate a significantly different health burden of NCDs, which varies with ethnicity and background. Data from NGOs providing them with medical assistance should be included in structuring public health interventions aimed at the prevention and treatment of NCDs. This could help to better allocate resources and address their health needs.

## Introduction

Migration to Europe from non-European countries has continued without interruptions over the last two decades. This has reshaped the population of most European countries, a part of which is now composed by heterogeneous groups of migrants, both documented and undocumented. This has an impact on many aspects of social services and public health^1^.

It has been shown that migrants from outside Europe already have, on arrival, a higher prevalence of HIV, tuberculosis and NCD^2–4^. These persons, especially if they are refugees and asylum seekers, can be frequently affected by mental health disturbances, the most common being post-traumatic stress disorder and depression^5^. If forced migrants have been victims of torture or exposed to potentially traumatic events, their risk of physical and mental health issues is further increased^6^.

These observations are in contrast to the previous hypothesis of a “healthy migrant effect”, which suggested that only young and healthy people would undertake the risks and uncertainty associated with migration from areas of poverty, famine or conflict^7^.

We now know that chronic conditions can affect migrants irrespective of the period they have been living in Europe^8^. Their health burden is compounded by factors which are not found in the same type of native patients. For instance, migrants can face many barriers in accessing healthcare due to linguistic, cultural and administrative problems, while being at high risk of NCDs and experiencing forced changes in their dietary habits^9^. Similarly to their countries of origin, many European Countries’ health systems are under financial pressures where drugs for NCDs can be unavailable or unaffordable^10^. Expenditure for prevention and treatment of NCDs is on the verge of sustainability and can add financial pressure to affected households^11^.

In the majority of European countries, undocumented migrants have limited access to healthcare, with the exception of emergency care, which is not an appropriate setting for continuous treatment of their chronic conditions^8^. Often their living conditions expose them to several risk factors for NCDs, and lack of stable financial income can limit their ability to afford investigations, treatment and follow-up for NCDs. Still, they are far from being exempt from these conditions^12^ and their health can deteriorate with duration of stay, as a consequence of difficulties described above and discrimination^13^. Conversely, it has been suggested that migrants with worsening chronic conditions might tend to return to their Countries of origin with advancing age, and this could result in falsely lower recorded mortality compared to native patients. This “remigration bias” has been questioned and is now considered to be not relevant^14^. However, it could have an effect on the reduced mortality observed in migrants in Italy, though studies available are usually carried out in samples of registered rather than undocumented migrants^15,16^.

In addition to this, social determinants of health appear to play a more significant role for migrants^13^, and they should be considered when designing a framework to understand migrants’ health needs on a national basis^17^.

Beside receiving under-treatment for their NCDs, undocumented migrants miss out on measures aimed at preventing complications from these chronic conditions. They are not included in any national healthcare database in Italy and, as a consequence, they cannot be reached by public health programs nor epidemiologic surveillance of the population. They remain a large but unfortunately almost invisible part of the population of all European countries. Therefore, the role of other providers of medical care such as NGOs becomes fundamental^18^. These organizations deliver health assistance to patients having no other chance of receiving it; moreover, some of them keep medical records of their clients. This makes it possible to have an insight into the more relevant health needs of these patients, which is very useful also because their health conditions may change over time as a consequence of a different demographic composition of this population, of factors related to the permanence in the host country, and social determinants.

This study was conducted in a large population of undocumented migrants living in Milan, Lombardy, the region with the highest proportion of these persons in Italy. Our aim was to describe potential changes over time with regards to the frequency of patients with NCDs, their demographics, clinical complexity, and healthcare requirements. Moreover, we evaluated whether undocumented migrants from different ethnicities demonstrate variations in their risk of being affected by NCDs, and compared this to Europeans living in similar socioeconomic conditions.

## Methods

### Study design

This was a retrospective observational study on the health status of undocumented migrants.

### Setting and participants

We evaluated the medical records of all the 53,683 patients seen during a 10-year period (2011–2020) at Opera San Francesco (OSF). This is the biggest NGO in Milan, Italy, which gives medical assistance for free to those from the poorest economic backgrounds, the majority of whom is represented by undocumented migrants. Clinics are run by doctors on a voluntary basis and all medical specialties are available. Patients come without need for reservation and are seen by an internist. If deemed necessary, the internist can refer the patient to a specialist or prescribe investigations. S/he can also prescribe medications, which are issued to the patient for free before leaving the facility.

### Variables

The following demographic characteristics gathered at the time of the first visit were collected: gender, age, nationality, year of first contact with OSF.

For each medical access, details about the prescribed drugs and one or more diagnoses were collected.

Medications were defined by their anatomical therapeutic code (ATC)^19^, while the diagnoses were collected following ICD-9 codes.

Each subject has an anonymous and univocal personal identification. All methods were performed in accordance with the relevant local guidelines and regulations.

### Identification of patients with NCD

To select patients with NCD, we used a list of chronic conditions containing both the ICD-9 code of that condition and the ATC code of the drugs used for its treatment (Table 1). All patients having either the ICD-9 code of an NCD or the ATC code of a drug used to treat it, or both, were considered as patients with NCD. For example, chronic deficiency of vitamin D is associated with ICD-9 code 268 and its pharmacological treatment with ATC code A11.

**Table 1:** ICD-9 and ATC codes used for defining selected non-communicable diseases (NCD).

Organs and systems	Disease	ICD-9 (from – to)	ATC
Chronic infectious diseases	Chronic infectious diseases	010–018.96	J04, J05
		042	
		070–070.9	
Cancer	All	140–239.9	L01
Diabetes	Diabetes	250.0	A10
Endocrine system diseases	Chronic endocrinopathies	240–246.9	H03, L02, G03
		255.0	
		255.4	
		256.4	
	Vitamin D deficiency	268	A11
	Lipid metabolism disorders	272–272.9	C10
	Gout	274–274.9	M04
	Obesity	278–278.8	A08
Blood diseases	Anaemias	280–285.9	B03
Mental diseases	All	290–315.9	N05, N06
Nervous system diseases	Neurodegenerative diseases	331–331.2	N04, N06D
		332–332.1	
	Pain	338	N02
	Epilepsies	338.4	N03
	Migraine	346–346.91	N02C
Sense organs diseases	Glaucoma	365–365.9	S01
Heart and vessels diseases	Chronic cardiovascular diseases	401–405.99	C01, C02, C07, C08, C09, B01
		410–414.9	
		427–427.9	
		428–428.9	
	Chronic venous system diseases	451–451.9	B01, C05
		454–454.9	
		455–455.9	
Respiratory system diseases	Respiratory system diseases	490–493.91	R03
Digestive system diseases	Digestive system diseases	535.4–535.6	A02, A03, A05, A07
		555	
		556–556.9	
Genitourinary system diseases	Chronic kidney diseases	585–586	A11, C03, B03
	Prostatic hyperplasia	600–601.9	G04
Skin diseases	Psoriasis	696.0–696.8	D05

### Statistical analysis

Categorical variables were summarized as absolute and relative frequencies. Given the unstable nature of this population of undocumented migrants, it is indeed difficult to be certain of their presence on the territory over a long period of time. Therefore, to avoid bias in the calculation of the percentage of NCDs, we stratified the count for each year from 2011 to 2020, using as a denominator only patients with at least one contact at the NGO during that year. So, for each year, the percentage of patients with NCD was calculated as the ratio between the distinct number of patients with NCD in that year (numerator) and the total number of distinct individuals seen at OSF (denominator). The annual distribution of individuals with NCD was further stratified by gender, age and geographical origin, by dividing the number of individuals with NCD in a specific stratum (e.g. male individuals) and the number of individuals belonging to the same strata seen at OSF in a specific year. We also calculated age-adjusted NCD rates in the considered categories using direct standardization with the age composition in the year 2011 as reference.

Clinical complexity was measured by evaluating other comorbidities affecting each patient with NCD. In particular, for each of the considered NCD, the following were measured: (1) the percentage of patients also affected by other NCDs; (2) the percentage of individuals who only have that NCD; and (3) the mean number of comorbidities in patients affected by that NCD.

Multivariate log-binomial regression models were used to assess the association between any (or specific) NCD disease and demographic characteristics. Models were adjusted by gender, age category (< 18, 18–39, 40–64 or ≥ 65) and geographical origin area (Europe, Africa, Asia, Latin America and other/missing) and year of first contact with the clinic. Results were summarized as Risk Ratios (RR) and 95% Confidence Intervals (CI). The Statistical Analysis System software (version 9.4; SAS Institute, Cary, NC) was used for the statistical analysis. For all hypotheses tested, two-tailed *P* values < 0.05 were considered significant.

### Ethics

This study was approved by the Ethics Committee of the University of Milano Bicocca. Protocol title: “Studio dell’evoluzione temporale delle NCD nella popolazione migrante irregolare ed italiana povera assistita presso un Ente Sanitario Caritativo di Milano tra il 2011 e il 2020”. Approval No. 676, 25 January 2???. This study was based on reviewing patients records and informed consent was obtained from all patients. All the data were completely and permanently anonymized.

## Results

Of the 53,683 clients having at least one contact with OSF in the period of the study, there were 29,650 males (55.2%) and 24,033 females (44.8%), though in recent years females have shown a tendency to outnumber males (data not shown). They were predominantly young adults, with a median age of 35 years. The majority of them were African (31.6%) and Latin American (31.1%); Europeans were 26.2% and Asians 11.1%.

In this population, 17,292 had at least one NCD. Their mean age was slightly older (median 40 years) than that of the general population and females were more than males (51.8% vs 48.2%). There were 647 minors (under 18 years of age) with at least one NCD (13.3% of all minors). In the elderly (over 65 years), this was instead equal to 1,086 (51.0% of over 65yo). Latin Americans were by far the most represented (40.8%). Europeans were 26.7%, Africans 22.4% and Asians 10%. Further details are available in Table 2.

**Table 2:** Socio-demographic characteristics of the total cohort of 53,683 subjects and of 17,292 subjects with non-communicable disease (NCD).

	NCD subjects	Overall subjects with at least one contact
	N (% of total)	
N	17,292 (100.0)	53,683 (100.0)
Age (at the first diagnosis/contact)		
< 18	647 (3.7)	4213 (11.6)
18–39	7,886 (45.6)	20,003 (55.0)
40–64	7,673 (44.4)	11,132 (30.6)
≥ 65	1,086 (6.3)	1043 (2.9)
Median (min–max)	40 (0–97)	35 (0–100)
Year of first NCD diagnosis/contact		
2011	2398 (13.9)	8609 (16.0)
2012	2032 (11.8)	6549 (12.2)
2013	1789 (10.3)	6202 (11.6)
2014	1550 (9.0)	5853 (10.9)
2015	1461 (8.4)	4993 (9.3)
2016	1482 (8.6)	4549 (8.5)
2017	1497 (8.7)	5001 (9.3)
2018	1459 (8.4)	4747 (8.8)
2019	2059 (11.9)	4444 (8.3)
2020	1565 (9.1)	2736 (5.1)
Sex		
Male	8328 (48.2)	29,650 (55.2)
Female	8964 (51.8)	24,033 (44.8)
Geographical origin		
Europe	4630 (26.7)	14,086 (26.2)
*Italy*	*755 (4.4)*	*3845 (7.2)*
*Western Europe*	*45 (0.3)*	*187 (0.3)*
*Eastern Europe*	*3830 (22.1)*	*10,054 (18.7)*
Africa	3871 (22.4)	16,923 (31.6)
*North Africa*	*2205 (12.8)*	*11,247 (21.0)*
*Sub-Sahran Africa*	*1666 (9.6)*	*5676 (10.6)*
Asia	1727 (10.0)	5940 (11.1)
*Central Asia*	*195 (1.1)*	*448 (0.8)*
*South Asia*	*1334 (7.7)*	*4866 (9.1)*
*Eastern Asia*	*113 (0.7)*	*317 (0.6)*
*Middle East*	*85 (0.5)*	*309 (0.6)*
Latin America	7058 (40.8)	16,706 (31.1)
Other/Missing	6 (0.0)	28 (0.1)

Significant values are in [italics].

**Figure 1:**
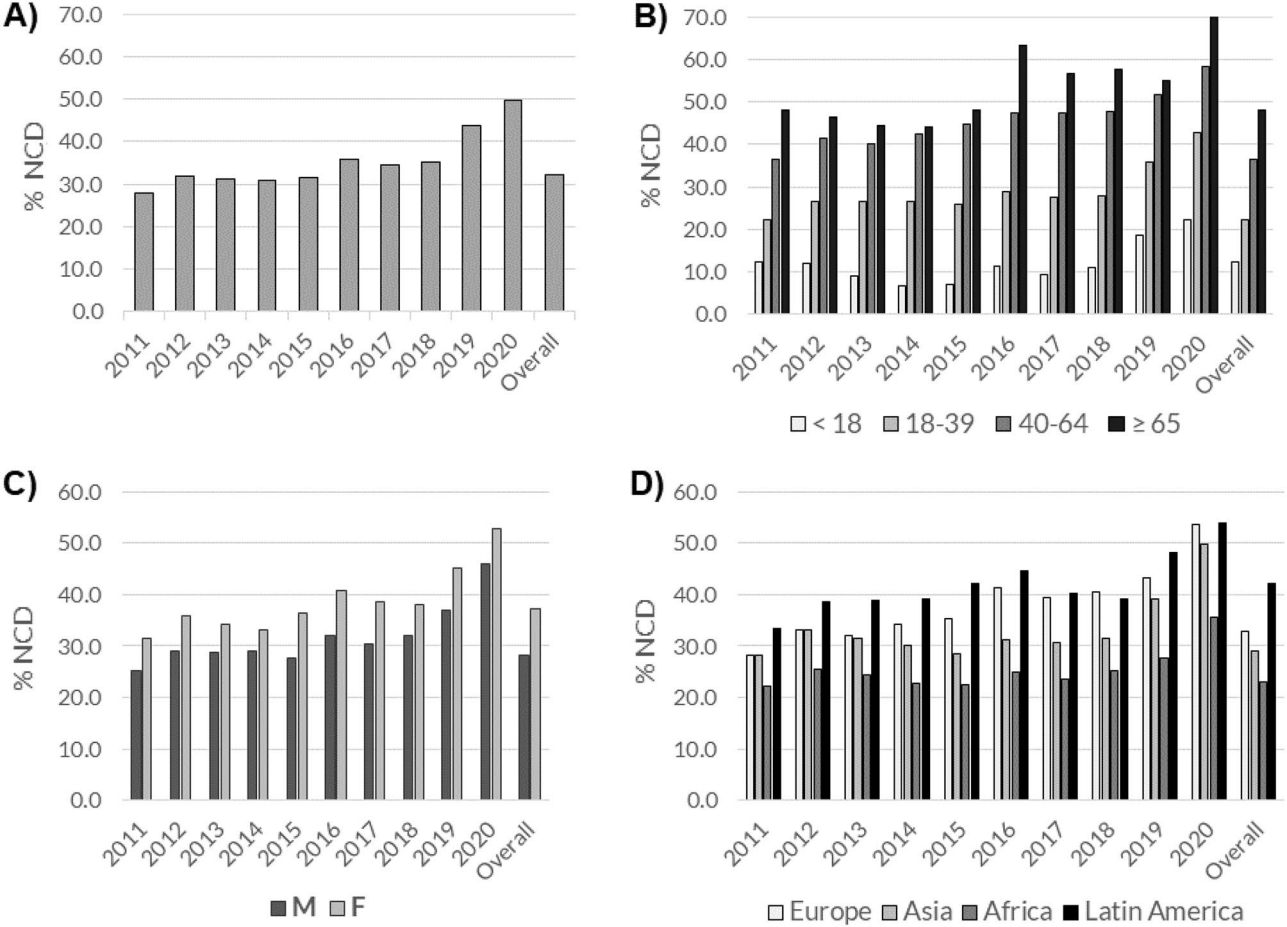
Annual distribution of subjects with non-communicable diseases (NCD) among overall (**A**) and stratified by age category (**B**), sex (**C**), and geographical origin (**D**).

As shown in Fig. 1 and in Supplementary Table 1 (with age-standardized value), the percentage of clients diagnosed as having at least one NCD increased steadily from 2011 to 2020. The increased percentage of patients with NCD was not due a generalized increase of the different NCD taken into consideration. Rather, it resulted from the fact that patients with some NCD increased, while those with other decreased. For example, patients with diabetes were 9% of all NCD patients in 2011 and 11.5% in 2020; those with cardiovascular diseases increased from 28.4 to 32.4%. In the same period, chronic respiratory diseases decreased from 10.8 to 6.8%, and mental health disorders from 26.2 to 22.1%.

The relative frequency and percentage of patients with NCD during the study period are shown in Table 3. Neurological diseases were the most frequent, though they were almost only represented by pain, i.e. ICD-9 code 338 and/or ATC class N02. Digestive system diseases were the second most represented diagnosis, affecting 39.4% of NCD patients. Cardiovascular diseases and mental health disorders were present in almost one third of patients, while the miscellaneous group of endocrine system diseases accounted for 21.2% of the diagnoses. This group did not include diabetes, which was diagnosed in 9.7% of NCD patients. Genitourinary diseases were present in 16% of NCD patients, with chronic kidney disease accounting for 12.6%.

**Table 3:** Distribution of non-communicable diseases (NCD).

NCD	N of patients	%
Chronic infectious diseases	227	1.3
Cancer	985	5.7
Diabetes	1669	9.7
Endocrine system diseases*	3671	21.2
*Neurodegenerative endocrinopathies*	*1189*	*6.9*
*Vitamin D deficiency*	*729*	*4.2*
*Lipid metabolism disorders*	*854*	*4.9*
*Gout*	*150*	*0.9*
*Obesity*	*1375*	*8*
Blood diseases	1071	6.2
Mental diseases	5082	29.4
Nervous system diseases	7026	40.6
*Neurodegenerative diseases*	*60*	*0.3*
*Pain*	*6581*	*38.1*
*Epilepsies*	*768*	*4.4*
*Migraine*	*311*	*1.8*
Sense organ diseases	2243	13
Heart and vessel diseases	5358	31
*Chronic cardiovascular diseases*	*4222*	*24.4*
*Chronic venous system diseases*	*2545*	*14.7*
Respiratory system diseases	1889	10.9
Genitourinary system diseases	2759	16
* Chronic kidney diseases*	*2187*	*12.6*
* Prostatic hyperplasia*	*711*	*4.1*
Skin diseases	561	3.2
TOTAL number of patients with at least one NCD	17,292	100

*Excluding diabetes.

Significant values are in [italics].

These percentages are referred to diagnoses, but a single patient might have more than one. This raises the problem of comorbidities, the analysis of which is presented in Fig. 1. In spite of their relatively young age, our NCD patients had usually three or more comorbidities, with only a smaller percentage being affected by only one NCD.

We then analyzed the drugs prescribed (and dispensed) to treat three of the most resource-consuming chronic conditions: cardiovascular diseases, mental health disorders and diabetes (Table 4). For the first group of conditions, anti-hypertensive medications, those for heart failure and aspirin were the most prescribed. For the second, benzodiazepines (various classes) were predominant, but also antidepressants and neuroleptics were used. Diabetic patients were treated predominantly with metformin and insulin glargine.

**Table 4:** Distribution of the ten most frequently prescribed medications used for the treatment of cardiovascular diseases, mental health disorders and diabetes.

ATC	Description	Frequency	% of all selected prescription of the group
Heart and vessel diseases			
C08CA01	Amlodipine	2123	11.87
B01AC06	acetylsalicylic acid	1828	10.22
C07AB07	Bisoprolol	1719	9.61
C09AA05	Ramipril	1554	8.69
C09AA02	Enalapril	1061	5.93
C09DA03	Valsartan and diuretics	700	3.91
C09BA05	Ramipril and diuretics	566	3.16
C09DA04	Irbesartan and diuretics	407	2.28
C07AB03	atenolol	402	2.25
C05AA10	Fluocinolone acetonide	367	2.05
Mental diseases			
N05BA06	Lorazepam	835	10.22
N05BA12	Alprazolam	764	9.35
N06AA09	Amitriptyline	758	9.28
N05BA08	Bromazepam	695	8.5
N05BA	Benzodiazepine derivatives	689	8.43
N06AB05	Paroxetine	650	7.95
N05AH03	Olanzapine	322	3.94
N06AX21	Duloxetine	287	3.51
N05AH04	Quetiapine	275	3.37
N06BX12	Acetylcarnitine	248	3.03
Diabetes			
A10BA02	Metformin	2121	37.93
A10AE04	Insulin glargine	664	11.87
A10BB12	Glimepiride	512	9.16
A10AB04	Insulin lispro	350	6.26
A10AB05	Insulin aspart	345	6.17
A10AB06	Insulin glulisine	269	4.81
A10AE05	Insulin detemir	233	4.17
A10BB09	Gliclazide	210	3.76
A10BD02	Metformin and sulfonylureas	200	3.58
A10BH01	Sitagliptin	92	1.65

The risk of being affected by of these NCD was higher in women than in men (RR = 0.88, 95% CI 0.86–0.90) and significantly increased with age (p for trend < 0.001). Moreover, ethnic origin also affected the relative risk. Compared to Europeans, Africans and Asians had a lower risk of having cardiovascular diseases (RR = 0.62, CI 0.58–0.67 and RR = 0.85, CI 0.78–0.92, respectively) and mental disorders (RR = 0.66, CI 0.61–0.71 and RR = 0.60, CI 0.54–0.67, respectively), while these risks were increased in Latin Americans (RR = 1.07, CI 1.01–1.13 and RR = 1.23, CI 1.19–1.26, respectively). Moreover, in migrants from Asia and Latin America the relative risk of diabetes was higher, compared to Europeans (RR = 1.68, CI 1.44–1.97 and RR = 1.39, CI 1.21–1.60, respectively) (Table 5).

**Table 5:** Multivariate risk ratios, along with 95% confidence intervals, estimated from a log-binomial regression model for evaluating the association between patients’ characteristics and non-communicable disease (NCD).

Compared groups		Any NCD	Cardiac	Mental	Diabetes
		17,292 with any NCD	5358 with cardiovascular diseases	5082 with mental diseases	1669 with diabetes
		VS	VS	VS	VS
		36,391 without NCD	36,391 without NCD	36,391 without NCD	36,391 without NCD
Age class (at first visit)	< 18	0.46 (0.43–0.50) ***	0.10 (0.09–0.10) ***	0.46 (0.41–0.53) ***	0.08 (0.03–0.21) ***
	18–39	1 ^Ref^	1 ^Ref^	1 ^Ref^	1 ^Ref^
	40–64	1.38 (1.34–1.41) ***	3.63 (3.41–3.86) ***	1.35 (1.28–1.42) ***	7.16 (6.26–8.18) ***
	> = 65	1.64 (1.57–1.71) ***	6.12 (5.66–6.61) ***	1.15 (1.00–1.32) ^ns^	not estimable
	*p* for trend	< .0001	< .0001	< .0001	< .0001
Sex	F	1 ^Ref^	1 ^Ref^	1 ^Ref^	1 ^Ref^
	M	0.88 (0.86–0.90) ***	0.84 (0.80–0.88) ***	0.80 (0.76–0.84) ***	1.08 (0.97–1.07) ^ns^
Geographical origin	Europe	1 ^Ref^	1 ^Ref^	1 ^Ref^	1 ^Ref^
	Africa	0.75 (0.72–0.77) ***	0.62 (0.58–0.67) ***	0.66 (0.61–0.71) ***	0.79 (0.68–0.92) *
	Asia	0.91 (0.84–0.91) **	0.85 (0.78–0.92) ***	0.60 (0.54–0.67) ***	1.68 (1.44–1.97) ***
	Latin America	1.23 (1.19–1.26) ***	1.07 (1.01–1.13) ***	1.18 (1.11–1.25) ***	1.39 (1.21–1.60) ***
	Other/Missing	0.69(0.35–1.44) ^ns^	1.26 (0.54–2.95) ^ns^	0.58 (0.16–2.19) ^ns^	1.40 (0.22–9.04) ^ns^

Models were also adjusted for year of first visit/prescription.

Models were adjusted by sex, age category, geographical origin and year of first contact with the clinic.

***p < 0.001; **p < 0.01; *p < 0.05; ^ns^ not significant; ^Ref^ Reference category.

## Discussion

This study in a large sample of undocumented migrants shows that they are frequently affected by NCDs and that the percentage of people with at least one chronic condition increased from 2011 to 2020. This is especially the case since 2016 and demonstrated an acceleration in 2019 and 2020. The risk of having an NCD increased with age and was higher in females. Finally, we evaluated the relative risk of being affected by a chronic condition in undocumented migrants of different geographical origin, compared to Europeans attending OSF and living in similar social and economic conditions. We believe this provides useful information, in consideration of the continuous flow of migrants from extra-European countries, having as a potential consequence a reshaping of the epidemiology of NCD in European countries.

Considering NCDs together, we observed that the relative risk is lower in people from Asia and Africa, and higher in Latin Americans. When we considered individually the three more relevant chronic diseases, this difference remained true for cardiovascular diseases and mental health disorders, but not for diabetes.

We also noted ethnicity related differences. For cardiovascular diseases, Europeans were at lower risk than Latin Americans and at higher risk than Africans and Asians. Both Latin Americans and Asians had an increased risk of diabetes.

Within the population of patients attending OSF clinics, the percentage of those with NCD has increased over the years. It is known that the burden of chronic conditions is high in patients attending health care facilities providing medical assistance to the poor^12,20^. It is also known that the burden of NCD is growing all over the world and, if this trend continues, NCD will have caused 52 million deaths by 2030, with NCD-related costs estimated to be US Dollars 47 trillion from 2010 to 2030^12,21^. The fact that the percentage of patients with chronic conditions has increased in our population of undocumented migrants could reflect this generalized tendency. However, for years 2019 and 2020 we have also to consider a potential role of the COVID-19 epidemic. In our case, the known association between COVID-19 morbidity and NCD^23^ does not seem to be the most important factor; rather, the fact that other NGOs were not operating during the pandemic could have induced more undocumented migrants with NCDs to seek medical help from OSF.

Interestingly, despite a growing percentage of patients with chronic conditions, their composition in terms of age, gender and ethnicity did not change significantly over the years, while the relative percentage of the various NCDs did. At present, our population of undocumented migrants seems to be relatively young, but with a high burden of NCDs and many comorbidities. The latter appear to be an important issue when evaluating their health conditions. To date, two studies, one in Switzerland^24^ and one in Spain^25^, have addressed this problem. The first was conducted in Geneva, one of the only 2 Swiss Cantons which have implemented primary care services within the public healthcare system for undocumented migrants. The other was carried out in the region of Aragon during 2011, at a time (2000–2012) when undocumented migrants too were allowed to access public healthcare services in Spain. Both are retrospective studies based on the analysis of electronic health records of the entire population offering an estimate of the prevalence of comorbidities. This is different in the two studies: the percentage of patients with comorbidities was 23% in women and 14% in men for the Swiss study versus 12% and 8% for the Spanish study. We cannot comment whether our results, which do not refer to prevalence in the general population, are in keeping with those of one of the above studies. They give only an idea of the percentage of patients with comorbidities within a group of undocumented migrants with NCD, though attention should be exerted when interpreting this observation since some coexisting conditions could be related to confounders. For example, this could well be the case of the association of blood and genitourinary diseases, the first being often represented by chronic anemia secondary to long-lasting menstrual losses.

Independently from the method used to evaluate comorbidity, in a context of such poverty, it is probably better to think of it as syndemics. The concept of a syndemic was developed by medical anthropologists in the nineties and refers to synergistic health problems affecting a population experiencing social and economic inequalities^26^. It entails the concept of multiple illnesses interacting clinically and biologically with each other and with the socio-economic and cultural environment^27^. This means, for instance, that a disease as diabetes can be complicated by the association with different other diseases, depending on the socio-economic conditions of the environment^28^. Therefore, for the population of this study we deem it more appropriate to think of multiple coexisting illnesses in terms of syndemics.

Nevertheless, we have already shown that the treatment of chronic conditions—either alone or associated with other diseases—is limited to pharmacological interventions not very different from those implemented for patients belonging to the general population^29,30^. This could be due to the fact that NGOs have very scarce possibilities to impact on social and economic determinants; therefore, other types of intervention are almost impossible.

When we analyzed specific chronic conditions, we found differences in the frequency of cardiovascular diseases among different ethnic groups. This is in agreement with previous studies, showing that significant differences exist in cardiovascular morbidity and mortality between migrants and natives^31^, and that these differences are not always in favor of the hosting population^32,33^.

In our population, mental health disorders too were found to vary in migrants arriving from different geographical areas, with Latin Americans having a higher relative risk, as well as for cardiovascular diseases. It is known that the prevalence of mental disorders depends on many factors and shows great variations also among various European countries. For example, depression prevalence has an approximately four times difference between the lowest-ranked and highest-ranked income Countries^34^.

The relative risk of diabetes was significantly higher in Asians and Latin Americans. This is in keeping with the observation that the frequency of type 2 diabetes is higher in migrants of all ethnicities and that they develop this disease at an earlier age than native Europeans^35^, though ethnic origin does not appear to be the only explanation^30^.

Our observations support the view that dealing with chronic diseases of undocumented migrants is becoming a priority issue in Europe, though it can be regarded as part of the larger problems of NCD prevention and treatment globally. Some of these diseases are now considered to be at least in part communicable, for example through social and economic conditions, viruses, urbanisation and industrialization, food deserts (i.e., areas with shortage of healthy food such as fresh fruit and vegetables) and intergenerational transmission, like for diabetes and obesity^33^. On this basis, it has been proposed to rename them “socially transmitted conditions” (STCs)^37^. This definition conveys also the difficulties we encounter in preventing and treating these conditions, which require addressing poverty and other social issues, pollution and other environmental problems, and opposition by strong economic interests. At present, the most effective strategies to prevent NCD are still based on individuals and their capacity to adhere to a healthy lifestyle^38^, but this is especially difficult with undocumented migrants for many obvious reasons. Moreover, as outlined above, they suffer many limitations in accessing medical treatment and follow-up for their chronic conditions. Finally, even the mitigating effect of social capital is reduced^39^. As a consequence, they experience worse health conditions and are at risk of developing other serious, yet preventable, health problems^40^.

We are aware that our study also has some limitations. For example, since it is impossible to know the exact number of undocumented migrants living in Lombardy over a certain period of time, we cannot give any data on the real prevalence of NCD in this population; the only information we have is the relative frequency of the various NCD in our sample of undocumented migrants. Moreover, as we have already stated, OSF is the biggest and best organized NGO providing medical assistance to undocumented migrants in Milan and this could generate a selection bias. Even more important is the fact that we have no conclusive information on how long our undocumented migrants have been living in Milan, though we know that they are not newly arrived. This could be critical to distinguishing between their previous and more recent lifestyles and their relative impact on the present illness. This is due to many difficulties, mainly the unwillingness of some of them to declare the exact moment of arrival, and the fact that some of them frequently move from a Country to another and therefore they can be exposed to different lifestyles. This is a limitation of the present study, but we are now trying to identify sufficiently reliable methods to define the exact time spent in our city by these persons, in order to provide a more complete picture in forthcoming studies.

With regards to the representativeness of our sample population, it should be noted that Lombardy represents an extremely attractive destination for migrants in search of better living conditions, since it is the Italian region where the most important Italian economic and industrial activities are concentrated.

The “Regional Observatory for Integration and Multi-Ethnicity” reports that 1,170,000 migrants currently live in Lombardy, a number corresponding to a quarter of the entire migrant population living in Italy. 153,400 (13%) of the migrants living in Lombardy are undocumented and they are mainly concentrated in the province of Milan (69,000, of which 44,000 in Milan)^41^. From these data it can therefore be inferred that the undocumented migrant community residing in Lombardy is highly representative of the entire Italian undocumented population.

The major point of strength of our study is the large sample of undocumented migrants, while many studies available in literature are focused on documented migrants. Indeed, a lot of work has been done by many authors, including Italians, with undocumented migrants, but mainly at the moment of their arrival, and we now know that their epidemiology is quite different^42,43^.

The first recommendation we can draw from the present study is that undocumented migrants should be taken into consideration by health policy makers in Europe. A distinction between newly arrived and resident undocumented migrants appears to be fundamental, since they have different health needs that should be addressed differently. These needs are different from those of undocumented migrants in prolonged settlements^44^.

At a public health level, the problem of NCDs risks to become the more resource-consuming for these patients as it already is for natives, therefore it deserves special attention. An interaction between NGOs and public healthcare systems would be extremely helpful and it is now time to take steps in this direction.

WHO indications and guidelines are of paramount importance to shape more comprehensive strategies of tackling NCDs in the entire population, though the WHO definition of NCD can be somehow limited^45^ and here the scope is expanded.

New technologies, such as telemedicine, could prove useful in monitoring NCDs in this very mobile part of the population and educational interventions could begin to have an impact on modifiable factors involved in the pathogenesis of NCDs, such as smoking and obesity.

## Conclusions

If the health issues of undocumented migrants are not addressed in a timely manner—especially NCDs—this could lead to their conditions deteriorating, more complications and further comorbidities. This would perpetuate a cycle of worsening life conditions, requiring more robust and resource-consuming interventions in the future. It is therefore necessary to implement public health interventions to cope with chronic diseases of undocumented migrants, especially diabetes, cardiovascular diseases and mental health disorders. Since these conditions are unevenly distributed in different ethnic groups, targeted interventions could be designed.

## Supplementary Information

Supplementary Information.

## Data Availability

Data files used for the present used can be requested to the corresponding author.
