# Supplementary material for: Characterizing non-communicable disease trends in undocumented migrants over a period of 10 years in Italy
Source: Sci Rep. 2023 May 8;13:7424. doi: 10.1038/s41598-023-34572-3 (PMC10167203; doi:10.1038/s41598-023-34572-3)
Supplement: Supplementary file 1 — Supplementary Information. [file 41598_2023_34572_MOESM1_ESM.docx]

**Supplementary Table 1.** Annual percentage of subjects with non-communicable diseases (NCD) in each considered group with raw and age-standardized value.

|  |  | **Overall** |  | **Age** | | | |  | **Sex** | |  | **Geographical origin** | | | |
| --- | --- | --- | --- | --- | --- | --- | --- | --- | --- | --- | --- | --- | --- | --- | --- |
|  |  |  |  | **<18** | **18-39** | **40-64** | **≥65** |  | **M** | **F** |  | **Europe** | **Asia** | **Africa** | **Latin America** |
| 2011 | raw | 27.9 |  | 12.5 | 22.2 | 36.4 | 48.1 |  | 25.2 | 31.6 |  | 28.3 | 28.1 | 22.2 | 33.3 |
|  | age-standardized | - |  |  |  |  |  |  | - | - |  | - | - | - | - |
| 2012 | raw | 32.0 |  | 12.0 | 26.7 | 41.4 | 46.3 |  | 29.2 | 35.9 |  | 33.1 | 33.1 | 25.4 | 38.5 |
|  | age-standardized | 32.1 |  |  |  |  |  |  | 29.2 | 36.2 |  | 33.0 | 33.2 | 25.3 | 38.9 |
| 2013 | raw | 31.0 |  | 9.1 | 26.7 | 40.2 | 44.3 |  | 28.8 | 34.2 |  | 32.0 | 31.4 | 24.3 | 38.7 |
|  | age-standardized | 31.5 |  |  |  |  |  |  | 28.6 | 35.1 |  | 30.1 | 35.2 | 24.0 | 38.9 |
| 2014 | raw | 30.7 |  | 6.6 | 26.6 | 42.3 | 44.2 |  | 28.9 | 33.1 |  | 34.1 | 30.1 | 22.7 | 39.0 |
|  | age-standardized | 32.1 |  |  |  |  |  |  | 29.2 | 35.4 |  | 36.8 | 30.8 | 22.8 | 39.8 |
| 2015 | raw | 31.5 |  | 7.2 | 25.8 | 44.8 | 48.1 |  | 27.8 | 36.3 |  | 35.2 | 28.4 | 22.4 | 42.0 |
|  | age-standardized | 32.6 |  |  |  |  |  |  | 27.6 | 38.4 |  | 37.5 | 28.6 | 22.0 | 43.5 |
| 2016 | raw | 35.9 |  | 11.5 | 28.8 | 47.4 | 63.1 |  | 32.0 | 40.6 |  | 41.3 | 31.1 | 24.8 | 44.6 |
|  | age-standardized | 36.0 |  |  |  |  |  |  | 31.3 | 42.3 |  | 41.9 | 31.1 | 24.3 | 46.1 |
| 2017 | raw | 34.4 |  | 9.5 | 27.7 | 47.3 | 56.7 |  | 30.4 | 38.6 |  | 39.4 | 30.8 | 23.6 | 40.3 |
|  | age-standardized | 35.1 |  |  |  |  |  |  | 30.9 | 40.5 |  | 40.9 | 30.6 | 24.0 | 42.0 |
| 2018 | raw | 35.2 |  | 11.1 | 28.0 | 47.8 | 57.7 |  | 32.1 | 38.1 |  | 40.4 | 31.5 | 25.2 | 39.2 |
|  | age-standardized | 35.6 |  |  |  |  |  |  | 32.0 | 39.7 |  | 41.4 | 30.8 | 24.6 | 40.8 |
| 2019 | raw | 41.3 |  | 18.5 | 35.9 | 51.8 | 55.0 |  | 36.9 | 45.3 |  | 43.4 | 39.1 | 27.6 | 48.2 |
|  | age-standardized | 41.7 |  |  |  |  |  |  | 36.8 | 46.3 |  | 44.7 | 37.6 | 31.5 | 49.1 |
| 2020 | raw | 49.6 |  | 22.2 | 42.8 | 58.2 | 71.5 |  | 45.9 | 52.7 |  | 53.5 | 49.8 | 35.6 | 54.0 |
|  | age-standardized | 48.5 |  |  |  |  |  |  | 43.9 | 52.7 |  | 51.1 | 49.1 | 34.4 | 53.7 |
